# Towards effectiveness of cell free DNA based liquid biopsy in head and neck squamous cell carcinoma

**DOI:** 10.1038/s41598-024-52031-5

**Published:** 2024-01-26

**Authors:** Ewelina Kowal-Wisniewska, Katarzyna Jaskiewicz, Anna Bartochowska, Katarzyna Kiwerska, Adam Ustaszewski, Tomasz Gorecki, Maciej Giefing, Jaroslaw Paluszczak, Malgorzata Wierzbicka, Malgorzata Jarmuz-Szymczak

**Affiliations:** 1 Institute of Human Genetics, Polish Academy of Sciences, Strzeszynska 32, 60-479 Poznan, Poland; 2 Department of Hematology and Bone Marrow Transplantation, Poznan University of Medical Sciences, Poznan, Poland; 3 Department of Otolaryngology and Laryngeal Oncology, Poznan University of Medical Sciences, Poznan, Poland; 4 Department of Tumor Pathology, Greater Poland Cancer Centre, Poznan, Poland; 5 Faculty of Mathematics and Computer Science, Adam Mickiewicz University, Poznan, Poland; 6 Department of Pharmaceutical Biochemistry, Poznan University of Medical Sciences, Poznan, Poland; 7 Faculty of Medicine Wroclaw, University of Science and Technology, Wroclaw, Poland

**Keywords:** Diagnosis, Prognosis, Genetic techniques, Biomarkers, Medical research, Molecular medicine, Oncology, Oral anatomy

## Abstract

Liquid biopsy is a minimally invasive procedure, that uses body fluids sampling to detect and characterize cancer fingerprints. It is of great potential in oncology, however there are challenges associated with the proper handling of liquid biopsy samples that need to be addressed to implement such analysis in patients’ care. Therefore, in this study we performed optimization of pre-analytical conditions and detailed characterization of cfDNA fraction (concentration, length, integrity score) in surgically treated HNSCC patients (n = 152) and healthy volunteers (n = 56). We observed significantly higher cfDNA concentration in patients compared to healthy controls (p < 0.0001) and a time dependent decrease of cfDNA concentration after tumor resection. Our results also revealed a significant increase of cfDNA concentration with age in both, healthy volunteers (p = 0.04) and HNSCC patients (p = 0.000002). Moreover, considering the multitude of HNSCC locations, we showed the lack of difference in cfDNA concentration depending on the anatomical location. Furthermore, we demonstrated a trend toward higher cfDNA length (range 35–10380 and 500–10380 bp) in the group of patients with recurrence during follow-up. In conclusion, our study provide a broad characterization of cfDNA fractions in HNSCC patients and healthy controls. These findings point to several aspects necessary to consider when implementing liquid biopsy in clinical practice including: (I) time required for epithelial regeneration to avoid falsely elevated levels of cfDNA not resulting from active cancer, (II) age-related accumulation of nucleic acids accompanied by less efficient elimination of cfDNA and (III) higher cfDNA length in patients with recurrence during follow-up, reflecting predominance of tumor necrosis.

## Introduction

Liquid biopsy fulfills the tempting vision of easy body fluid sampling to detect unique cancer fingerprints. The first report concerning cell free DNA (cfDNA) in blood samples was published over 70 years ago by Mandel and Metais^1^. In turn, the prerequisite for liquid biopsy as a diagnostic tool has been achieved in 1977 when Leon et al. proved that cfDNA is present at higher concentrations in cancer patients’ serum/plasma in comparison to healthy controls^2^. It has been further demonstrated that the cfDNA fraction is enriched in DNA released by tumor cells during apoptosis, necrosis or active secretion. This fraction of cfDNA is known as circulating tumor DNA (ctDNA)^3–5^. It has been proven that macrophages which phagocytosed necrotic or apoptotic cells release short digested DNA fragments in the case of necrotic cells^6^. Another factor differentiating between normal and tumor-derived cfDNA is the length of DNA fragments. It has been shown that tumor-derived cfDNA is shorter than this derived from normal cells^7^. Besides DNA concentration and fragment length, an alternative to characterize cfDNA for diagnostic purposes is the integrity score (IS). IS described as the ratio of concentration of longer to shorter DNA fragments in body fluids is an increasingly reported parameter described in liquid biopsy^8,9^. These cfDNA characteristics opened the avenue to apply liquid biopsy as a minimally invasive method to detect genetic and epigenetic alterations underlying the presence of the malignant disease. However, to ensure the highest quality of the performed diagnostic analysis, the significance of well optimized pre-analytical conditions needs to be highlighted.

The conditions of applied procedures that should be kept uniformly during analysis are related to: type of specimen (plasma or serum), type of blood collection tubes, time of blood processing, blood processing parameters, plasma/serum storage before cfDNA isolation, cfDNA extraction method, cfDNA quantification and finally the storage conditions^10,11^.

The identification of key parameters of cfDNA sample quality and the standardization of the cfDNA samples collection procedure will greatly increase the reliability of the findings reported in the literature, allow comparison of the results between studies and reinforce prospects of clinical implementation of liquid biopsy. Consistently, based on these assumptions, the aim of the performed study was the optimization of the protocol for blood sample donations and comprehensive characterization of cfDNA fraction in HNSCC patients taken before the surgery and after tumor resection to monitor the course of the disease in patients’ follow-up.

## Results

### Clinical characteristics of study groups

Plasma samples derived from patients at the time of disease/recurrence diagnosis (before surgery) were marked as the first donation (FD). 137 patients were diagnosed with primary tumor (PTu) and 15 with recurrent tumor (RTu) (Table S1, Fig. S1). After diagnosis, all patients were treated surgically, and 73 of them were subsequently treated with adjuvant radiotherapy or radiochemotherapy. Data concerning further treatment of 17 patients were not available. For all patients, the detailed clinicopathologic features such as tumor location, TNM staging (according 8th edition), and grading were collected and summarized in Tables 1 and 2. Data concerning the HPV status of tumor samples are unavailable. First blood donation was collected during ongoing tumor growth after the patient’s hospital admission, but before the surgical intervention. After the surgery, additional blood sample/s was/were collected during control visits at the scheduled time intervals described later. The subsequent blood sampling for patients treated with adjuvant radiotherapy or radiochemotherapy took place at least 16 weeks after the end of radiotherapy, with exception of 8 donations that were performed earlier. After completing the treatment, based on current oncological status, the patients were classified as cancer free or with tumor recurrence (two patients recurred twice). Overall, 186 blood samples of subsequent donations were obtained from patients classified as cancer free and 14 samples were derived from patients having a recurrence at that moment.

**Table 1:** Study group characteristics.

First donation n = 152	T					N					M		G				
	T1	T2	T3	T4	Tx	N0	N1	N2	N3	Nx	M0	NA	G0	G1	G2	G3	NA
Primary tumors (PTu) n = 137	n = 18	n = 58	n = 30	n = 31	n = 0	n = 93	n = 18	n = 25	n = 1	n = 0	n = 136	n = 1	n = 1	n = 29	n = 91	n = 10	n = 6
Reccurent tumors (RTu) n = 15	n = 1	n = 2	n = 2	n = 9	n = 1	n = 11	n = 0	n = 1	n = 0	n = 3	n = 15	n = 0	n = 0	n = 0	n = 13	n = 1	n = 1

**Table 2:** Sample subgroups depending on the tumor location at the time of first and subsequent donations.

	PTu/RTu n = 166	PTu n = 137	RTu n = 29
Group 1			
jaw, nose, nasal cavity, paranasal sinuses	n = 9	n = 7	n = 2
Group 2			
oral cavity, tongue, floor of the mouth, tongue and base of the oral, tongue and sublingual gland	n = 43	n = 37	n = 6
Group 3			
lip	n = 1	n = 1	n = 0
Group 4			
hypopharynx, hypopharynx and larynx	n = 6	n = 4	n = 2
Group 5			
larynx	n = 101	n = 84	n = 17
Group 6			
oropharynx, palatine tonsil	n = 6	n = 4	n = 2

Based on the clinical course of disease, samples from the first donation were classified by experienced ENT clinicians [A.B and M.W] into following subgroups: [I] first donation from patients free of cancer longer than two years (n = 96); [II] first donation from patients free of cancer, with follow-up shorter than two years (n = 18); [III] first donation from patients who developed recurrence after surgery—during follow up (n = 19); [IV] first donation from patients at the time of cancer recurrence (n = 15) (Fig. S1). The course of disease of 4 patients was unclear, so they were excluded from further study. Part of the analyses were performed on the study group consisted of all samples collected during ongoing carcinogenesis including [I] first PTu donations (n = 137), [II] first RTu donations at the time of disease recurrence (n = 15), [III] subsequent donations at the moment of disease recurrence (n = 14), altogether 166 cfDNA samples were used. Depending on the tumor location, samples were divided into six subgroups. Group 3 was excluded from the analyses because only one patient was classified into this group. Detailed information is presented in Table 2.

### cfDNA concentration

The usefulness of cfDNA concentration assessment for HNSCC detection and patients characterization was investigated. To describe the dynamics of cfDNA concentration changes during the course of the disease, samples taken from patients at different time points after tumor resection were included.

**Figure 1:**
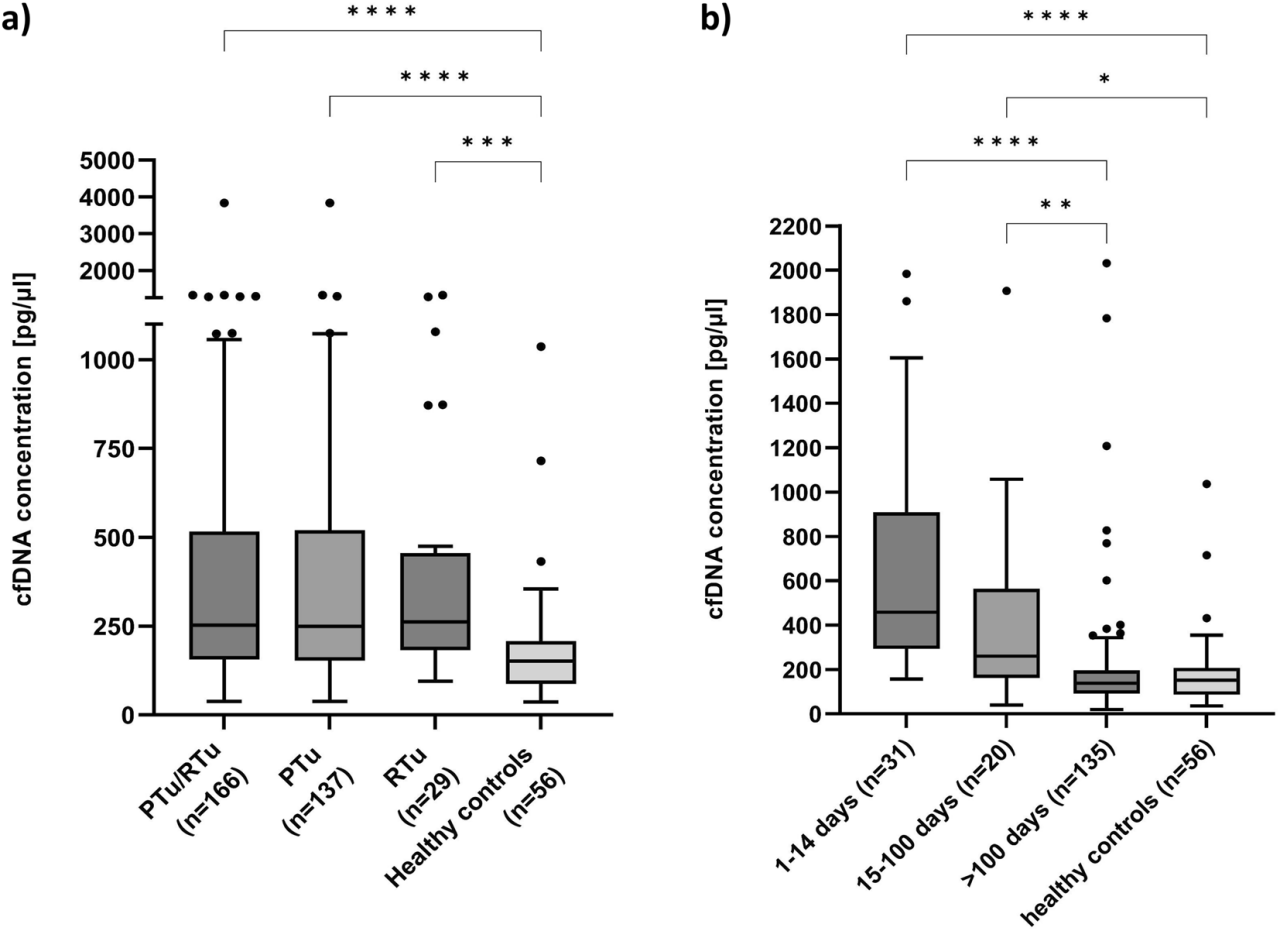
Concentration of cfDNA (range 35–250 bp) isolated from plasma of patients and healthy controls. (**a**) At the time of diagnosis of primary (PTu) and recurrent (RTu) tumors, as well as healthy controls. Kruskal–Wallis Rank Sum Test followed by post hoc Dunn's test was performed. PTu/RTu (n = 166), RTu (n = 29), PTu (n = 137), healthy controls (n = 56). (**b**) At different time points after tumor resection and healthy controls. Kruskal–Wallis Rank Sum Test followed by post hoc Dunn's test was performed. 1–14 days (n = 31), 15–100 days (n = 20), above 100 days (n = 135), healthy controls (n = 56).

The median concentration of cfDNA (range 35–250 bp) for samples collected during ongoing carcinogenesis (n = 166) was 258.51 pg/μl and was significantly higher (p < 0.0001) compared to healthy controls 151.92 pg/μl as presented in Fig. 1a. This difference remained, when patients with primary tumors (n = 137) 255.63 pg/μl vs controls (p < 0.0001) as well as when patients with recurrent tumors (n = 29) 261.38 pg/μl vs controls (p = 0.0006) were considered Fig. 1a. No significant difference between primary tumors and recurrent tumors was observed, but the median cfDNA concentration was higher in the latter (Fig. 1a).

Taking into account the repair/regeneration processes taking place in the body after surgery, as well as postoperative treatment, subsequent donations were then divided into three subgroups depending on the time between the surgery and blood sample collection: (I) 1–14 days, n = 31; (II) 15–100 days, n = 20 and (III) > 100 days, n = 135. The division into subgroups was proposed by experienced ENT specialists, assuming that average length of hospitalization after surgery is two weeks and the time necessary for epithelial regeneration is around 12 weeks. The median concentration of cfDNA in the established subgroups was as follows: (1–14 days, n = 31) 458.48 pg/μl; (15–100 days, n = 20) 259.70 pg/μl; (above 100 days, n = 135) 138.26 pg/μl.

Significantly higher cfDNA concentration level was found in subgroup I (458.48 pg/μl) and subgroup II (259.70 pg/μl) compared to healthy controls (p < 0.0001 and p = 0.0168, respectively). It should be emphasized that cfDNA concentration 100 and more days after tumor resection (138.26 pg/μl) is similar to healthy controls (p > 0.9999) (Fig. 1b).

**Figure 2:**
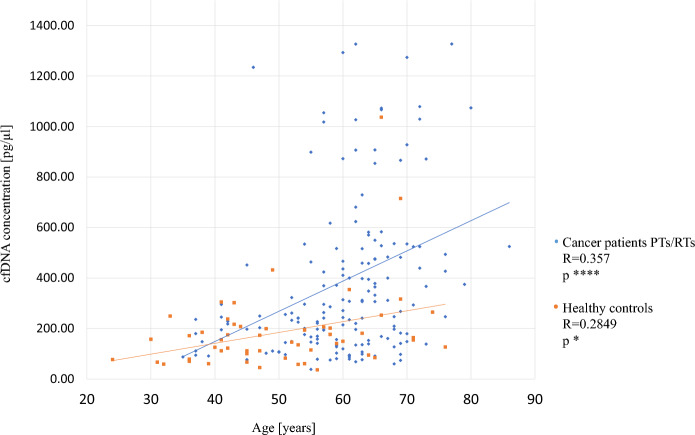
Correlation of cfDNA concentration in range 35–250 bp with age at donation (Spearman's rank correlation). Blue dots—cancer patients (n = 166); Orange dots—healthy controls (n = 56).

To evaluate whether age at donation had influence on cfDNA concentration, the correlation between both parameters was calculated for cancer patients as well as for healthy controls. There was a significant tendency observed that cfDNA concentration increases with age (p = 0.000002, r = 0.357 and p = 0.04, r = 0.2849 respectively) (Fig. 2). In healthy controls this tendency is not as strong as in cancer patients.

**Figure 3:**
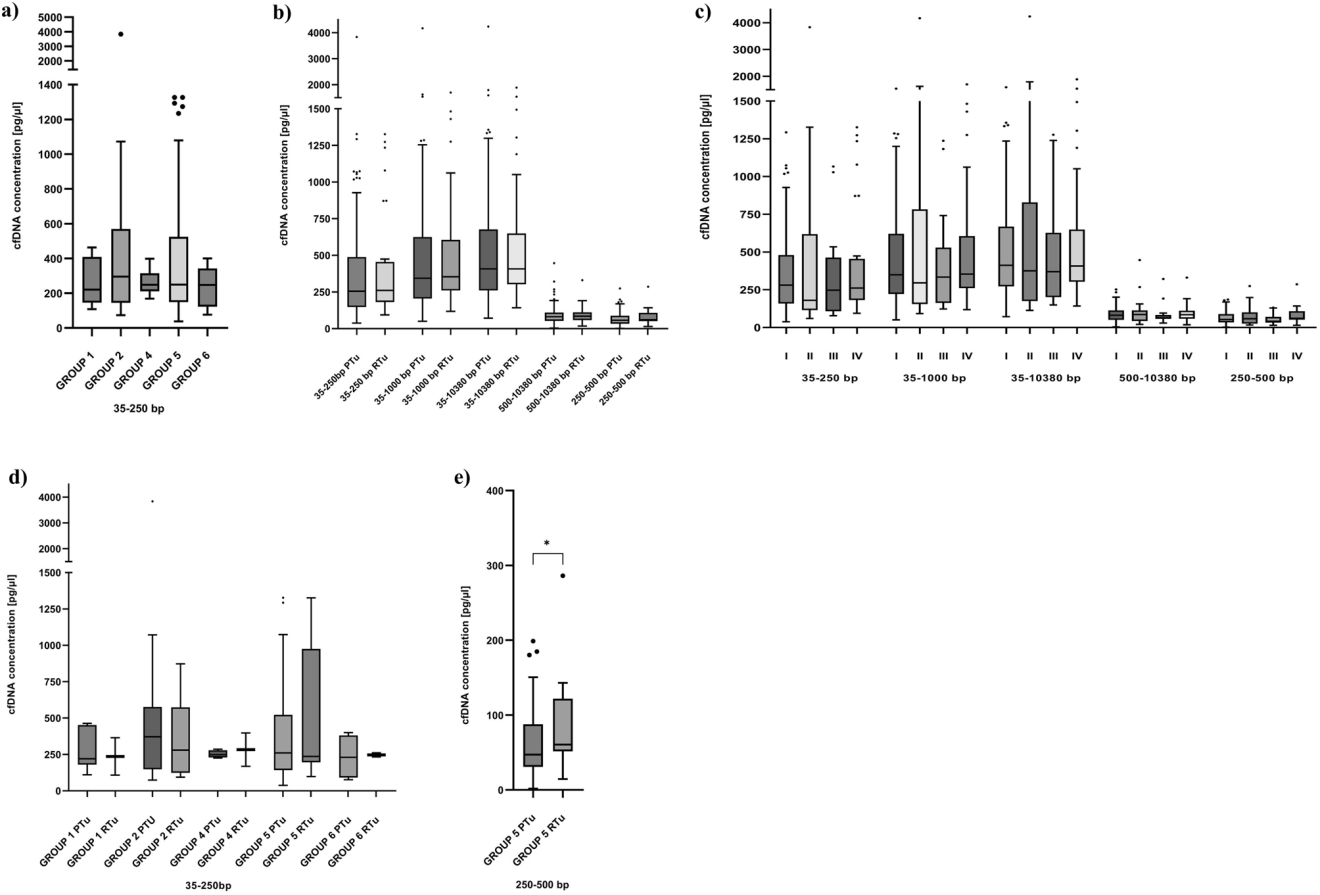
Concentration of cfDNA in HNSCC patients depending on selected variables. (**a**) CfDNA concentration in range 35–250 bp depending on the tumor location during ongoing tumor growth: group 1 (n = 9), group 2 (n = 43), group 4 (n = 6), group 5 (n = 101), group 6 (n = 6). Kruskal–Wallis Rank Sum Test followed by post hoc Dunn's test was performed. (**b**) Concentration of cfDNA in different ranges of length depending on the tumor type: primary (PTu, n = 137), recurrent (RTu, n = 29). The Mann–Whitney, two-sided test was performed. (**c**) Concentration of cfDNA in different ranges of length depending on the course of disease: (I) donation at the time of primary tumor diagnosis from patients free of cancer longer than two years (n = 96); (II) donation at the time of primary tumor diagnosis from patients free of cancer with follow-up shorter than two years (n = 18); (III) donation at the time of primary tumor diagnosis from patients with recurrence in follow-up (n = 19); (IV) donation at the time of recurrence diagnosis (n = 29). Kruskal–Wallis Rank Sum Test followed by post hoc Dunn's test was performed. (**d**) Concentration of cfDNA in range 35–250 bp depending on tumor location and the course of disease: group 1 PTu (n = 7), RTu (n = 2); group 2 PTu (n = 37), RTu (n = 6); group 4 PTu (n = 4), RTu (n = 2); group 5 PTu (n = 84), RTu (n = 17); group 6 PTu (n = 4), RTu (n = 2). The Mann–Whitney, two-sided test was performed. (**e**) Concentration of cfDNA in range 250–500 bp in larynx cancers, PTu (n = 84), RTu (n = 17). The Mann–Whitney, two-sided test was performed.

We also searched for possible correlations between cfDNA concentration, tumor location, and other clinical features described below. No significant differences in cfDNA concentration depending on tumor location was found (Table S2). It should be mentioned here that in the range 35–250 bp, the highest values of the median cfDNA concentration were observed in patients with cancers localized in the oral cavity (group 2; 295.39 pg/μl) compared to other locations, where median cfDNA concentration was approximately 248.12 pg/μl (Fig. 3a). Moreover, no significant difference in concentration of cfDNA isolated from patients diagnosed with primary tumors (n = 137) versus cfDNA concentrations at the moment of disease recurrence (n = 29) was observed concerning different ranges of cfDNA length (Fig. 3b).

To evaluate whether cfDNA concentration could predict clinical course of disease, a comparison of cfDNA concentration in different ranges of length was assessed for the following four groups: [I] donation at the time of primary tumor diagnosis from patients free of cancer longer than 2 years (n = 96); [II] donation at the time of primary tumor diagnosis from patients free of cancer with follow-up shorter than 2 years (n = 18); [III] donation at the time of primary tumor diagnosis from patients with recurrence in follow-up (n = 19); [IV] donation at the time of recurrence diagnosis (n = 29) (Fig. 3c). No significant difference in cfDNA concentration between analyzed subgroups was observed.

In addition, we performed comparisons of cfDNA concentrations in subgroups depending on tumor location and type of the disease (primary/recurrence) and several trends, albeit not significant in the concentration of cfDNA were observed (Table S2, Fig. S2). In range 35–250 bp, only a trend toward higher median cfDNA concentration in primary tumors compared to recurrent tumors in the case of larynx cancers and cancers localized in the oral cavity was observed (Fig. 3d). The opposite trend was observed in groups representing other locations (Fig. 3d). For larynx cancer, significantly higher cfDNA concentration of longer cfDNA fragments (250–500 bp) in patients with recurrent tumors compared to primary tumors was observed (p = 0.039) (Fig. 3e).

CfDNA concentration was also assessed depending on tumor location and T parameter (T1/T2 and T3/T4) according to the TNM classification. Similarly, only trends with no significant differences in cfDNA concentration were observed between these groups (Table S2, Fig. S3).

### cfDNA length

Mean cfDNA length in the group of patients and healthy controls was assessed, considering the clinical course of the disease. Mean cfDNA lengths were assessed in the following ranges: 35-250 bp, 35-1000 bp, 500-10380 bp, and 35-10380 bp.

**Figure 4:**
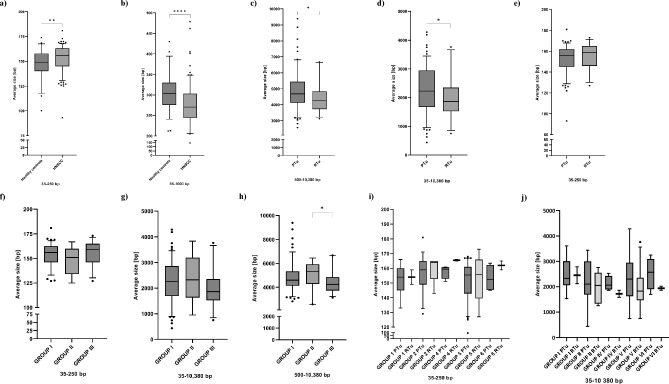
Average size of cfDNA in different length ranges. (**a**) 35–250 bp and (**b**) 35–1000 bp in cancer patients (n = 166) and healthy controls (n = 56). The Mann–Whitney, two-sided test was performed. (**c**) 500–10380 bp, (**d**) 35–10380 bp, (**e**) 35–250 bp depending on clinical course of disease including patients with primary (PTu, n = 137) and recurrent (RTu, n = 29) tumors. The Mann–Whitney, two-sided test was performed. (**f**) 35–250 bp, (**g**) 35–10380 bp, (**h**) 500–10380 bp depending on clinical course of disease including patients with primary tumors without recurrence (group I, n = 114), primary tumors with reccurence (group II, n = 19) and patients with reccurent tumors (group III, n = 29). Kruskal–Wallis Rank Sum Test followed by post hoc Dunn's test was performed. (**i**) 35–250 bp, (**j**) 35–10380 bp depending on tumor location and clinical course of disease including patients with primary tumors and with reccurent tumors: group 1 PTu (n = 7), RTu (n = 2); group 2 PTu (n = 37), RTu (n = 6); group 4 PTu (n = 4), RTu (n = 2); group 5 PTu (n = 84), RTu (n = 17); group 6 PTu (n = 4), RTu (n = 2). The Mann–Whitney, two-sided test was performed.

First, the average length of cfDNA between cancer patients and healthy controls was compared (Table S3). The difference in median cfDNA length in the range 35–250 bp was significant in cancer patients (156 bp) compared to healthy controls (149 bp) (p = 0.0061) (Fig. 4a) and in cancer patients (270 bp) compared to healthy controls (304 bp) in the range 35–1000 bp (p < 0.0001) (Fig. 4b). After that, cfDNA length was compared between patients diagnosed with primary (n = 137) and recurrent tumors (n = 29). We found significantly higher median cfDNA length in ranges: 500–10380 bp (p = 0.017) and 35–10380 bp (p = 0.0435) in patients with primary tumors (4679 bp; 2224 bp respectively) compared to patients with recurrence (4245 bp; 1864 bp respectively) (Fig. 4c,d) and a trend toward higher length in range 35–250 bp in patients with recurrent tumors (159 bp) compared to primary tumors (156 bp) (Fig. 4e). To further assess whether there are differences depending on the course of the disease, samples were divided into following groups: [I] first donation from patients without recurrence n = 114 (first donation from patients free of cancer longer than 2 years (n = 96) plus first donation from patients free of cancer, with follow-up shorter than 2 years (n = 18)); [II] first donation at the time of primary tumor diagnosis from patients with recurrence in the follow-up (n = 19); [III] first and subsequent donation at the time of recurrence (n = 29). Median cfDNA length in the range 35–250 bp was the lowest in the group II (151 bp) compared to group I (156 bp) and III (159 bp) (Fig. 4f). In range 35–10380 bp and 500–10380 bp median cfDNA length for group II (2328 bp and 5302 bp, respectively) was the highest compared to group I (2252 bp and 4599 bp, respectively) and group III (1864 bp, p = 0.18 and 4245 p = 0.035 bp, respectively) (Fig. 4g,h). There was no significant difference between median cfDNA length and tumor location in all analyzed ranges of lengths, however, in the range 35–250 bp higher median cfDNA length for recurrent tumors is observed for all analyzed groups (Fig. 4i). Furthermore, in the range 35–10380 bp lower cfDNA length is observed in the case of recurrent tumors for all included tumor location (Fig. 4j).

### IS score

**Figure 5:**
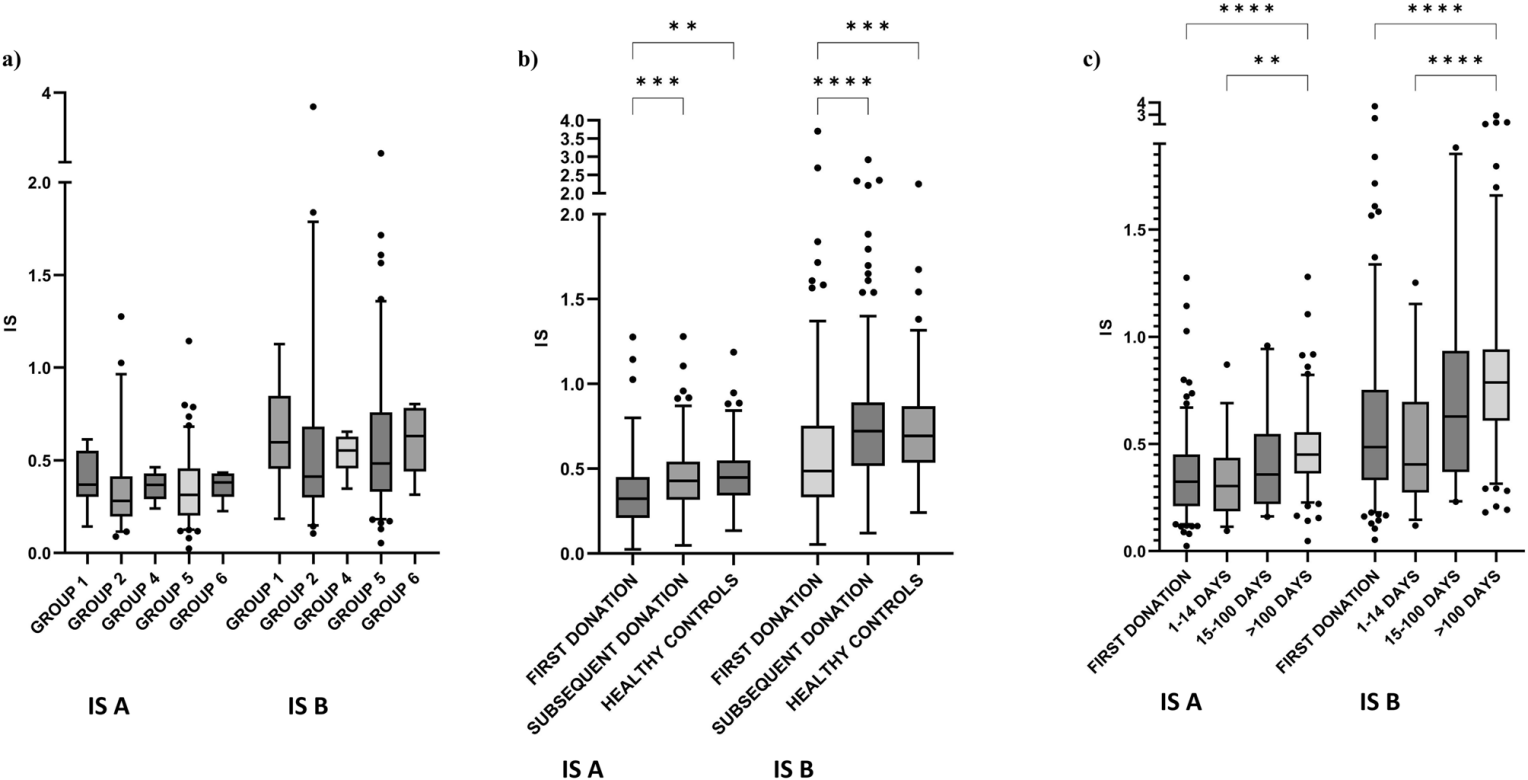
IS A and IS B values depending on different clinical features including: (**a**) tumor location group 1 (n = 9), group 2 (n = 43), group 4 (n = 6), group 5 (n = 101), group 6 (n = 6); (**b**) type of donation: first donation (n = 152), subsequent donation from patients without recurrence (n = 186) and healthy controls (n = 56); (**c**) time relapsed since the surgery 1–14 days (n = 31), 15–100 days (n = 20), above 100 days (n = 135) and first donation (n = 152). Kruskal–Wallis Rank Sum Test followed by post hoc Dunn's test was performed.

To assess the ratio of longer and shorter cfDNA fragments the integrity score (IS) of cfDNA was assessed. Integrity score was assessed as the ratio between cfDNA concentration in ranges 250–1000 bp (IS A) or 250–10380 (IS B) and 35–250 bp. IS value close to 1 means equal concentration of longer and shorter cfDNA fragments, IS value below 1 means the overrepresentation of shorter cfDNA fragments (35–250 bp) and IS value above 1 means the overrepresentation of longer cfDNA fragments. A comparison of IS A and IS B values for different tumor locations revealed no significant differences, however a trend toward lower median IS values was observed in the cancers localized in the oral cavity (group 2) and larynx (group 5) in comparison to other locations (Fig. 5a). Significant differences in IS A and IS B values for first donations (median ratios 0.32 and 0.49) compared to subsequent donations (median ratios 0.43 and 0.72) (p = 0.0001 and p < 0.0001, respectively) as well as first donations compared to healthy controls (median ratios 0.45 and 0.69) (p = 0.0012 and p = 0.0003, respectively) were found (Fig. 5b). The IS A and IS B values for subsequent donations and healthy controls are very similar. Our data indicate a significant influence of the time between surgery and blood collection on IS A and IS B values. It is further reflected by differences between subsequent donations depending on the time elapsed since the surgery showing that overrepresentation of shorter cfDNA fragments decreases over time after surgery (Fig. 5c).

## Discussion

Recent years have brought new possibilities for the use of liquid biopsy to detect tumors and monitor the course of disease in a minimally invasive manner^12^. Elevated levels of cfDNA are not observed only in cancers but are also characteristic for inflammatory diseases, including liver cirrhosis, hepatitis, systemic lupus erythematosus, or rheumatoid arthritis^13^. Therefore, characterization of cfDNA fraction which is composed of DNA released by normal and tumor cells is mandatory for any type of tumor^10,14^. Despite many advantages, liquid biopsy based assays require well characterized and optimized pre-analytical and analytical steps to ensure the highest quality and reproducibility of the performed analysis. In our study, we optimized the above-mentioned steps in HNSSC patients and compared the obtained results among patients subgroups and healthy controls.

Firstly, we used peripheral blood as a universal material, although saliva is also being considered for HNSCC oral cavity cancers^15^. Secondly, to avoid high background of DNA released by leukocytes during clotting, which may dilute ctDNA fraction, we used plasma samples instead of serum^16–18^. Moreover, from the variety of available blood collection tubes, S-Monovette EDTA tubes were used, in line with the latest recommendation for blood processing and maintaining proper preanalytical conditions^16,19^. Noteworthy, there are also tubes dedicated for liquid biopsy studies available, designed to preserve cfDNA despite delayed blood processing^16^. Hemolysis could lead to a high background of genomic DNA, which negatively affects subsequent analysis. In our experience, it can be prevented by gentle handling of blood samples and plasma isolation within 2 h after donation. Two-step hemolysis assessment is essential for discarding samples in which the results may be biased by the presence of abnormally high background of genomic DNA. Therefore, we pointed out pre-analytical sample handling and properly design schedule for subsequent donation as a critical procedural steps.

In our study, we showed a significant difference in total cfDNA level between cancer patients and healthy controls, confirming the enrichment of cfDNA with a fraction of DNA released by tumor cells^20^. However, the results obtained herein did not show any significant differences in cfDNA concentration between primary and recurrent tumors. Therefore, it seems that in patients with HNSCC, liquid biopsy along with evaluation of cfDNA level can be used to confirm the presence of a tumor, especially since cfDNA level is fairly constant regardless of the type of the lesion (primary/recurrent). Another important finding of our study is the necessity to consider time required for epithelial regeneration, when the decrease of cfDNA level after surgery is expected. To avoid false results (elevated levels of cfDNA that are not the result of active cancer), we established the 100-day cut-off to test cfDNA level after tumor resection. Changes in cfDNA concentration observed in this study justify our approach and confirm the previous findings that after such a time, only minimal background of cfDNA released by regenerating cells is observed and that cfDNA levels in cancer free patients return to levels observed in healthy controls^20–22^. There were some additional unique observations made in our study, concerning patients’ age, primary tumor location and its size. We observed a trend toward an age-related increase of cfDNA levels in both cancer patients and healthy controls, albeit stronger in cancer patients. As aging is defined as chronic, low-grade inflammation, we assume that this increase in cfDNA concentration with age results from age-related accumulation of both metabolites and nucleic acids^23^, accompanied by less efficient elimination of cfDNA with age^24^. In our study, due to the age homogeneity of the cancer and control groups, this trend did not affect the obtained results. Changes in cfDNA level in cancer patients may be related to high heterogeneity of HNSCC from different anatomical locations. In order to further analyze these differences, we divided patients into 6 groups according to the anatomical locations. Some trends in cfDNA concentration depending on the location of the tumor were observed (the highest cfDNA concentration in the range of 35–250 bp in group of cancers localized in oral cavity), however the results are not significant. We hypothesize that this observation is related to HPV infections in the pathogenesis of cancers localized in oral cavity. However, this is in contrast to the results presented by Mazurek et al., who pointed out that HPV infection does not influence cfDNA concentration in OPSCC^25^. Considering the use of liquid biopsy in clinical practice, the lack of significant difference in concentration of cfDNA isolated from patients diagnosed with HNSCC with different anatomical locations seems to be extremely important. Thus, liquid biopsy may be universally used during HNSCC diagnosis. The biggest, although not significant differences in median cfDNA concentration depending on type of tumor lesion (primary/recurrence) were observed in larynx cancers group and oral cavity cancers group compared to other location. What is however important, significantly higher concentration of longer cfDNA fragments (250–500 bp) was confirmed in recurrent larynx tumors group compared to the primary tumors (PTu < RTu). Interestingly, there were no significant differences in median cfDNA concentration depending on tumor size (T) observed in our study indicating that liquid biopsy can be used at any clinical stage of the disease. There is an ongoing discussion regarding the relationship between cfDNA concentration and clinical parameters. In addition, proportion of cfDNA released by tumor cells varies between patients^13^. In many cancers cfDNA concentration positively correlates with both tumor size^18,26^ and disease stage^12,25^. A comprehensive study involving patients diagnosed 18 different types of cancer (including HNSCC) with defined stage of disease revealed that ctDNA concentration increased with disease stage, similarly to percentage of patients with detectable levels of ctDNA. The fraction of patients with localized disease and detectable ctDNA level is considerably lower compared to patients diagnosed with metastatic cancer^12^. On the other hand, as was demonstrated by Jung et al., the effect of stage, size and location on cfDNA concentration vary depending on the location and type of neoplasm^27^. Moreover, the literature data show divergent results from studies concerning the same tumor type^27^. Concerning HNSCC, Mazurek et al. reported higher level of total cfDNA in patients diagnosed with N2–N3 compared to N0–N1 disease, as well as in patients with stage IV in comparison to stages I–III^25^. These discrepancies may be caused by many factors, other than stage of the disease and tumor size influencing the amount of cfDNA released into bloodstream^28^. Silvoniemi et al. revealed positive correlation between maximum VAF and tumor burden measured with metabolic imaging (FDG-PET/CT) in HNSCC patients, confirming the influence of tumor growth potential on detection of somatic variants in ctDNA^29^.We further decided to characterize the cfDNA fractions observed in HNSCC patients and compare the results to non-cancer individuals taking into account also mean cfDNA length and the integrity factor. The average length of cfDNA was determined to be 166 bp long, which corresponds to the length of DNA wrapped around histone proteins and forming nucleosomes together with the linker DNA^4,7,18,30,31^. In our study, a statistically significant higher average cfDNA length in the range of 35–250 bp was detected in HNSCC patients (156 bp) compared to healthy controls (149 bp). In contrast, in the range of 35–1000 bp, a statistically significant higher average cfDNA length was observed in healthy controls (304 bp) compared to HNSCC (270 bp). Presented results may suggest both the influence of the loss of epigenetic control in cancer cells resulting in chromatin relaxation, as well as the co-occurrence in different proportions of mechanisms by which cfDNA is released in normal and cancer cells^32^.

Our results also revealed significantly higher cfDNA length in the range 500–10380 bp and 35–10380 bp in patients with primary tumors compared to patients with recurrent tumors. Moreover, we showed that in the group of patients with primary tumors and recurrence in follow-up higher cfDNA length was observed in ranges 500-10380 bp and 35–10380 compared to patients with primary tumors without recurrence in follow-up. It has been proven that macrophages which phagocytosed necrotic cells release short digested DNA fragments into circulation^6^, however exceeding of phagocytic capacity of macrophages could result in the presence of longer cfDNA fragments in circulation^6,33^. Therefore, we assumed that these longer than 1000 bp DNA fragments were released into circulation via necrosis^3^, suggesting predominance of tumor necrosis in the group of patients with primary tumors and recurrence in follow-up. It was already confirmed for renal cell carcinoma, that tumor necrosis may be used as an important predictor for recurrence^34^. Last we analyzed the IS factor which allows to indicate in which length of DNA fragments the cfDNA fraction is enriched. IS factor adopted in our study is inversely proportional to concentration of cfDNA in range 35–250 bp. We demonstrated slightly lower IS values in the case of cancers localized in larynx and oral cavity which is in line with the results showing higher concentration of cfDNA in range 35–250 bp in these groups of patients. Moreover, we observed significantly lower IS value for cancer patients compared to healthy controls and patients after tumor resection, which, in terms of using liquid biopsy in clinical practice, can be extremely useful. We have also shown that IS A and IS B values for subsequent donations increase with time after surgery, reaching levels similar to healthy controls 100 days after surgery. Similar observations were repeated by Ellinger et al. in prostate cancer patients^35–37^. Despite the described findings, our study has several limitations. The group of patients who developed recurrence during the course of the disease or who were diagnosed with recurrence was small compared to the group of patients without recurrence, therefore this analysis should be verified in a larger cohort of recurrent patients. In addition, in order to observe the influence of tumor location on cfDNA parameters the study groups should be equinumerous, but in our study, most of the samples came from patients diagnosed with cancer localized in the larynx and oral cavity. To conclude this study presents a wide characteristic of cfDNA fraction in HNSCC patients. We revealed higher cfDNA concentration in cancer patients compared to healthy controls. Additionally, the implemented study design allowed for the determination of a 100-day cut-off for subsequent donation after tumor resection for liquid biopsy, which minimalizes the risk of false positive results in patients free of cancer. In addition, proposed IS score showed significant differences between cancer patients and healthy controls and subsequent donation. Our results indicate that despite the very heterogeneous nature of HNSCCs, the concentration, size, and IS score remain sufficiently coherent to open the perspective of application of liquid biopsy in the clinic. However, it seems that in HNSCC patients diagnostic procedures based on liquid biopsy like cfDNA concentration, length, and IS score, should rather be used to confirm the presence of the tumor but not to predict recurrence.

## Materials and methods

### Study group

This single-center (Department of Otolaryngology and Laryngological Oncology, Poznan University of Medical Sciences, Poland), prospective, case–control study was conducted between 2014 and 2020. Overall, 152 patients (128 males and 24 females) with surgically treated HNSCC tumor at different stage of progression, as well as 56 healthy volunteers (48 males and 8 females), were included. The median age for the HNSCC group was 62, whereas for the control group 48 (Table S1). The study protocol was approved by the Institutional Review Board at Poznan University of Medical Sciences in accordance with the Declaration of Helsinki (289/12, 505A/15, 448/17, 910/17, 721/18, 549/21, 235/14, 502/15, 1156/18, 970/22). Each subject gave written informed consent to participate in the study and donated blood sample at least once.

### Plasma isolation and hemolysis assessment

Blood samples were collected to S-Monovette EDTA tubes and plasma was isolated within 2 h from blood collection. Centrifugation was performed twice (1. 1200*g*, 10 min; 2. 2000*g*, 10 min, room temperature) to separate plasma and remove cells and cell debris. Thereafter, a two-step hemolysis assessment was performed. First was a visual inspection, where all red-colored plasma samples were discarded. Second, the measurement of plasma absorbance at 414 nm was carried out to detect the presence of free oxyhemoglobin. All samples with absorbance above 0.3 were qualified as hemolyzed and excluded from further analysis^38^. Plasma samples which passed two step hemolysis assessment were divided into 1 ml portions and stored at − 80 °C.

### cfDNA isolation, quantity, and quality assessment

Prior to cfDNA isolation plasma samples were thawed on ice and centrifuged 16000*g*, at 4 °C for 10 min, to remove cell debris. CfDNA was isolated using QIAamp MinElute ccfDNA Kit (Qiagen, Hilden, Germany) according to the manufacturer’s protocol, involving pre-concentration of circulating nucleic acids onto magnetic beads and a cleanup on columns. Immediately after cfDNA isolation, quantity and quality assessment was performed using Agilent High Sensitivity DNA Kit (Agilent, Waldbronn, Germany), which was developed for sizing and quantification of DNA fragments and DNA smears in the 50–7000 bp size range down to pg/µl sensitivity. To cover the entire population of cfDNA, lower (35 bp) and upper (10380 bp) markers were defined as boundary ranges of analysis. The concentration of cfDNA was assessed in the following DNA length ranges: 35–10380 bp (total cfDNA), 35–250 bp (cfDNA derived mostly from apoptotic cells) as well as 250–500 bp, 500–10380 bp and 35–1000 bp (cfDNA derived from apoptotic and necrotic cells). Ultimately, cfDNA concentration was calculated per 1 ml of plasma used for isolation.

### Statistical analysis

For the statistical analysis two tests depending on the analyzed data sets were used. The statistical differences between two independent groups without normal data distribution (Shapiro–Wilk test) were evaluated using non-parametric Mann–Whitney test (GraphPad Prism 9). The multiple comparisons were performed using Kruskal–Wallis Rank Sum Test followed by post hoc Dunn's test (GraphPad Prism 9). Significance level was defined as 0.05. The box plots (Figs. 1, 3, 4, 5) were prepared with the use of GraphPad Prism 9 version. *P*-values < 0.05*, *p*-values < 0.01**, *p*-values < 0.001*** and *p*-values < 0.0001****. The correlation between cfDNA concentration and age was assessed using Spearman’s rank correlation test.

### Supplementary Information

Supplementary Information.

## Data Availability

The datasets generated during and analysed during the current study are available from the corresponding author on reasonable request.

## Abbreviations

cfDNA: cell free DNA
FD: first donation
PTu: primary tumor
RTu: recurrent tumor
ENT clinicians: Ear, Nose and Throat clinicians
IS: integrity score
HNSCC: head and neck squamous cell carcinoma
