# Supplementary material for: Towards effectiveness of cell free DNA based liquid biopsy in head and neck squamous cell carcinoma
Source: Sci Rep. 2024 Jan 26;14:2251. doi: 10.1038/s41598-024-52031-5 (PMC10817923; doi:10.1038/s41598-024-52031-5)
Supplement: Supplementary file 1 — Supplementary Information. [file 41598_2024_52031_MOESM1_ESM.docx]

| Table S1 HNSCC patients characteristics |  |  |
| --- | --- | --- |
|  |  |  |
| Characteristics |  | n(%) |
|  |  |  |
| Total patients (First donation ) |  | 152 |
| Males |  | 128 (84.2) |
| Females |  | 24 (15.8) |
|  |  |  |
| Healthy controls |  | 56 |
| Males |  | 48 (85.7) |
| Females |  | 8 (14.3) |
|  |  |  |
| Patients age (years) |  |  |
| mean |  | 61 |
| median |  | 62 |
| range |  | 35-86 |
|  |  |  |
| Healthy controls age (years) |  |  |
| mean |  | 51 |
| median |  | 48 |
| range |  | 24-76 |
|  |  |  |
| First donation (Total patients) |  | 152 |
| Primary tumors (PTu) |  | 137 (90.1) |
| PTu, recurrence free longer than two years |  | 96 (70.1) |
| PTu, recurrence free, follow-up shorter than two years |  | 18 (13.1) |
| PTu, recurrence in follow-up |  | 19 (13.9) |
| PTu, NA |  | 4 (2.9) |
| Recurrent tumors (RTu) |  | 15 (9.9) |
|  |  |  |
| Subsequent donations |  | 200 |
| At the time of cancer free |  | 186 (93) |
| At the time of recurrence |  | 14 (7) |
|  |  |  |
|  |  |  |
|  |  |  |

| Table S2 HNSCC patients characteristics concerning cfDNA concentration | | | |  |  |  |
| --- | --- | --- | --- | --- | --- | --- |
|  |  |  |  |  |  |  |
| Range | Donation at the time of ongoing carcinogenesis[pg/μl ] |  | N=166.00 |  | * Ptu+Rtu |  |
| 35-250 bp | mean |  | 390.45 |  |  |  |
|  | median |  | 258.51 |  |  |  |
|  | range |  | 38.25-3 835.13 |  |  |  |
|  |  |  |  |  |  |  |
| 35-1 000 bp | mean |  | 493.55 |  |  |  |
|  | median |  | 349.27 |  |  |  |
|  | range |  | 50.74-4 174.48 |  |  |  |
|  |  |  |  |  |  |  |
| 35-10 380 bp | mean |  | 549.34 |  |  |  |
|  | median |  | 407.75 |  |  |  |
|  | range |  | 71.9-4 239.07 |  |  |  |
|  |  |  |  |  |  |  |
| 250-500 bp | mean |  | 68.36 |  |  |  |
|  | median |  | 59.30 |  |  |  |
|  | range |  | 1.74-286.25 |  |  |  |
|  |  |  |  |  |  |  |
| 500-10 380 bp | mean |  | 91.36 |  |  |  |
|  | median |  | 81.25 |  |  |  |
|  | range |  | 3.97-446.73 |  |  |  |
|  |  |  |  |  |  |  |
|  | Donation at the time of ongoing carcinogenesis PTu [pg/μl ] |  | N=137 |  |  |  |
| 35-250 bp | mean |  | 383.81 |  |  |  |
|  | median |  | 255.63 |  |  |  |
|  | range |  | 38.25-3 835.13 |  |  |  |
|  |  |  |  |  |  |  |
| 35-1 000 bp | mean |  | 484.13 |  |  |  |
|  | median |  | 344.32 |  |  |  |
|  | range |  | 50.74-4 174.48 |  |  |  |
|  |  |  |  |  |  |  |
| 35-10 380 bp | mean |  | 539.94 |  |  |  |
|  | median |  | 407.93 |  |  |  |
|  | range |  | 71.9-4 239.07 |  |  |  |
|  |  |  |  |  |  |  |
| 250-500 bp | mean |  | 66.25 |  |  |  |
|  | median |  | 57.60 |  |  |  |
|  | range |  | 1.75-274.78 |  |  |  |
|  |  |  |  |  |  |  |
| 500-10 380 bp | mean |  | 90.47 |  |  |  |
|  | median |  | 81.14 |  |  |  |
|  | range |  | 3.97-446.73 |  |  |  |
|  |  |  |  |  |  |  |
|  | Donation at the time of ongoing carcinogenesis RTu [pg/μl ] |  | N=29 |  |  |  |
| 35-250 bp | mean |  | 421.84 |  |  |  |
|  | median |  | 261.38 |  |  |  |
|  | range |  | 94.34-1 326.39 |  |  |  |
|  |  |  |  |  |  |  |
| 35-1 000 bp | mean |  | 538.06 |  |  |  |
|  | median |  | 354.33 |  |  |  |
|  | range |  | 118.31-1 701.35 |  |  |  |
|  |  |  |  |  |  |  |
| 35-10 380 bp | mean |  | 593.70 |  |  |  |
|  | median |  | 407.46 |  |  |  |
|  | range |  | 142.89-1 890.46 |  |  |  |
|  |  |  |  |  |  |  |
| 250-500 bp | mean |  | 78.34 |  |  |  |
|  | median |  | 61.03 |  |  |  |
|  | range |  | 14.43-286.25 |  |  |  |
|  |  |  |  |  |  |  |
| 500-10 380 bp | mean |  | 95.73 |  |  |  |
|  | median |  | 84.71 |  |  |  |
|  | range |  | 17.86-330.92 |  |  |  |
|  |  |  |  |  |  |  |
|  | Healthy controls [pg/μl ] |  | N=56 |  |  |  |
| 35-250 bp | mean |  | 182.55 |  |  |  |
|  | median |  | 151.92 |  |  |  |
|  | range |  | 36.17-1 036.93 |  |  |  |
|  |  |  |  |  |  |  |
| 35-1 000 bp | mean |  | 262.72 |  |  |  |
|  | median |  | 223.13 |  |  |  |
|  | range |  | 48.68-1 388.93 |  |  |  |
|  |  |  |  |  |  |  |
| 35-10 380 bp | mean |  | 301.96 |  |  |  |
|  | median |  | 258.83 |  |  |  |
|  | range |  | 60.11-1 469.12 |  |  |  |
|  |  |  |  |  |  |  |
| 250-500 bp | mean |  | 48.28 |  |  |  |
|  | median |  | 39.19 |  |  |  |
|  | range |  | 6.65-225.73 |  |  |  |
|  |  |  |  |  |  |  |
| 500-10 380 bp | mean |  | 71.12 |  |  |  |
|  | median |  | 61.17 |  |  |  |
|  | range |  | 15.23-237.45 |  |  |  |
|  |  |  |  |  |  |  |
|  |  |  |  |  |  |  |
|  | Subsequent donation [pg/μl ] |  | N=186.00 |  | ** patients were free of cancer |  |
| 35-250 bp | mean |  | 298.01 |  |  |  |
|  | median |  | 169.88 |  |  |  |
|  | range |  | 19.85-2 031.62 |  |  |  |
|  |  |  |  |  |  |  |
| 35-1 000 bp | mean |  | 395.65 |  |  |  |
|  | median |  | 254.18 |  |  |  |
|  | range |  | 36.25-250.41 |  |  |  |
|  |  |  |  |  |  |  |
| 35-10 380 bp | mean |  | 447.22 |  |  |  |
|  | median |  | 292.95 |  |  |  |
|  | range |  | 50-2 631.74 |  |  |  |
|  |  |  |  |  |  |  |
| 250-500 bp | mean |  | 63.91 |  |  |  |
|  | median |  | 48.21 |  |  |  |
|  | range |  | 7.3-388.07 |  |  |  |
|  |  |  |  |  |  |  |
| 500-10 380 bp | mean |  | 87.75 |  |  |  |
|  | median |  | 72.28 |  |  |  |
|  | range |  | 17.52-380.35 |  |  |  |
|  |  |  |  |  |  |  |
|  |  |  |  |  |  |  |
|  | Subsequent donation [pg/μl ]  1-14 days after surgery |  | N=31.00 |  |  |  |
| 35-250 bp | mean |  | 665.35 |  |  |  |
|  | median |  | 458.48 |  |  |  |
|  | range |  | 156.92-1 983.98 |  |  |  |
|  |  |  |  |  |  |  |
| 35-1 000 bp | mean |  | 843.65 |  |  |  |
|  | median |  | 628.09 |  |  |  |
|  | range |  | 242.68-2 502.41 |  |  |  |
|  |  |  |  |  |  |  |
| 35-10 380 bp | mean |  | 914.97 |  |  |  |
|  | median |  | 706.14 |  |  |  |
|  | range |  | 308.72-2 623.14 |  |  |  |
|  |  |  |  |  |  |  |
| 250-500 bp | mean |  | 122.76 |  |  |  |
|  | median |  | 90.91 |  |  |  |
|  | range |  | 19.25-388.07 |  |  |  |
|  |  |  |  |  |  |  |
| 500-10 380 bp | mean |  | 127.07 |  |  |  |
|  | median |  | 108.81 |  |  |  |
|  | range |  | 35.48-271.44 |  |  |  |
|  |  |  |  |  |  |  |
|  | Subsequent donation [pg/μl ]  15-100 days after surgery |  | N=20.00 |  |  |  |
| 35-250 bp | mean |  | 416.36 |  |  |  |
|  | median |  | 259.70 |  |  |  |
|  | range |  | 39.14-1 906.75 |  |  |  |
|  |  |  |  |  |  |  |
| 35-1 000 bp | mean |  | 537.45 |  |  |  |
|  | median |  | 354.82 |  |  |  |
|  | range |  | 76.63-2 373.39 |  |  |  |
|  |  |  |  |  |  |  |
| 35-10 380 bp | mean |  | 614.14 |  |  |  |
|  | median |  | 428.53 |  |  |  |
|  | range |  | 112.82-2 631.74 |  |  |  |
|  |  |  |  |  |  |  |
| 250-500 bp | mean |  | 79.72 |  |  |  |
|  | median |  | 64.14 |  |  |  |
|  | range |  | 13.73-344.64 |  |  |  |
|  |  |  |  |  |  |  |
| 500-10 380 bp | mean |  | 118.99 |  |  |  |
|  | median |  | 99.18 |  |  |  |
|  | range |  | 43.83-380.35 |  |  |  |
|  |  |  |  |  |  |  |
|  | Subsequent donation [pg/μl ]  >100 days after surgery |  | N=135.00 |  |  |  |
| 35-250 bp | mean |  | 196.13 |  |  |  |
|  | median |  | 138.26 |  |  |  |
|  | range |  | 19.85-2 031.62 |  |  |  |
|  |  |  |  |  |  |  |
| 35-1 000 bp | mean |  | 271.77 |  |  |  |
|  | median |  | 197.89 |  |  |  |
|  | range |  | 36.25-2 366.25 |  |  |  |
|  |  |  |  |  |  |  |
| 35-10 380 bp | mean |  | 315.09 |  |  |  |
|  | median |  | 238.44 |  |  |  |
|  | range |  | 50-2 455.51 |  |  |  |
|  |  |  |  |  |  |  |
| 250-500 bp | mean |  | 48.05 |  |  |  |
|  | median |  | 38.84 |  |  |  |
|  | range |  | 7.30-2 456.83 |  |  |  |
|  |  |  |  |  |  |  |
| 500-10 380 bp | mean |  | 74.10 |  |  |  |
|  | median |  | 66.04 |  |  |  |
|  | range |  | 17.52-204.68 |  |  |  |
|  |  |  |  |  |  |  |
|  | Donation at the time of ongoing carcinogenesis at different tumor locations [pg/μl ] |  |  |  |  |  |
|  | GROUP I |  | N=9.00 |  |  |  |
| 35-250 bp | mean |  | 262.65 |  |  |  |
|  | median |  | 220.25 |  |  |  |
|  | range |  | 108.11 -463.44 |  |  |  |
|  |  |  |  |  |  |  |
| 35-1 000 bp | mean |  | 362.96 |  |  |  |
|  | median |  | 301.50 |  |  |  |
|  | range |  | 163.35-728.23 |  |  |  |
|  |  |  |  |  |  |  |
| 35-10 380 bp | mean |  | 417.99 |  |  |  |
|  | median |  | 351.75 |  |  |  |
|  | range |  | 189.74-872.53 |  |  |  |
|  |  |  |  |  |  |  |
| 250-500 bp | mean |  | 67.81 |  |  |  |
|  | median |  | 48.16 |  |  |  |
|  | range |  | 34.21-168.91 |  |  |  |
|  |  |  |  |  |  |  |
| 500-10 380 bp | mean |  | 87.53 |  |  |  |
|  | median |  | 79.91 |  |  |  |
|  | range |  | 30.03-252.03 |  |  |  |
|  |  |  |  |  |  |  |
|  | GROUP II |  | N=43.00 |  |  |  |
| 35-250 bp | mean |  | 479.33 |  |  |  |
|  | median |  | 295.39 |  |  |  |
|  | range |  | 73.86 -3 835.13 |  |  |  |
|  |  |  |  |  |  |  |
| 35-1 000 bp | mean |  | 591.18 |  |  |  |
|  | median |  | 447.90 |  |  |  |
|  | range |  | 115.45-4 174.48 |  |  |  |
|  |  |  |  |  |  |  |
| 35-10 380 bp | mean |  | 653.35 |  |  |  |
|  | median |  | 557.51 |  |  |  |
|  | range |  | 121.56-4 239.07 |  |  |  |
|  |  |  |  |  |  |  |
| 250-500 bp | mean |  | 74.75 |  |  |  |
|  | median |  | 71.50 |  |  |  |
|  | range |  | 11.31-274.78 |  |  |  |
|  |  |  |  |  |  |  |
| 500-10 380 bp | mean |  | 99.27 |  |  |  |
|  | median |  | 85.83 |  |  |  |
|  | range |  | 3.97-446.73 |  |  |  |
|  |  |  |  |  |  |  |
|  | GROUP IV |  | N=6.00 |  |  |  |
| 35-250 bp | mean |  | 262.39 |  |  |  |
|  | median |  | 248.35 |  |  |  |
|  | range |  | 168.44-397.70 |  |  |  |
|  |  |  |  |  |  |  |
| 35-1 000 bp | mean |  | 352.53 |  |  |  |
|  | median |  | 329.42 |  |  |  |
|  | range |  | 246.43-493.33 |  |  |  |
|  |  |  |  |  |  |  |
| 35-10 380 bp | mean |  | 398.07 |  |  |  |
|  | median |  | 384.22 |  |  |  |
|  | range |  | 272.74-535.73 |  |  |  |
|  |  |  |  |  |  |  |
| 250-500 bp | mean |  | 60.91 |  |  |  |
|  | median |  | 61.18 |  |  |  |
|  | range |  | 52.79-71.1 |  |  |  |
|  |  |  |  |  |  |  |
| 500-10 380 bp | mean |  | 74.78 |  |  |  |
|  | median |  | 70.24 |  |  |  |
|  | range |  | 49.36-115.94 |  |  |  |
|  | GROUP V |  | N=101.00 |  |  |  |
| 35-250 bp | mean |  | 381.87 |  |  |  |
|  | median |  | 249.53 |  |  |  |
|  | range |  | 38.25-1 326.93 |  |  |  |
|  |  |  |  |  |  |  |
| 35-1 000 bp | mean |  | 483.60 |  |  |  |
|  | median |  | 344.09 |  |  |  |
|  | range |  | 50.74-1 701.35 |  |  |  |
|  |  |  |  |  |  |  |
| 35-10 380 bp | mean |  | 537.86 |  |  |  |
|  | median |  | 394.77 |  |  |  |
|  | range |  | 71.9-1 890.93 |  |  |  |
|  |  |  |  |  |  |  |
| 250-500 bp | mean |  | 67.17 |  |  |  |
|  | median |  | 56.24 |  |  |  |
|  | range |  | 1.75-286.25 |  |  |  |
|  |  |  |  |  |  |  |
| 500-10 380 bp | mean |  | 89.61 |  |  |  |
|  | median |  | 81.30 |  |  |  |
|  | range |  | 4.65-330.92 |  |  |  |
|  |  |  |  |  |  |  |
|  | GROUP VI |  | N=6.00 |  |  |  |
| 35-250 bp | mean |  | 238.55 |  |  |  |
|  | median |  | 247.07 |  |  |  |
|  | range |  | 76.49-399.90 |  |  |  |
|  |  |  |  |  |  |  |
| 35-1 000 bp | mean |  | 322.03 |  |  |  |
|  | median |  | 340.87 |  |  |  |
|  | range |  | 101.56-490.26 |  |  |  |
|  |  |  |  |  |  |  |
| 35-10 380 bp | mean |  | 369.54 |  |  |  |
|  | median |  | 384.25 |  |  |  |
|  | range |  | 113.35-572.9 |  |  |  |
|  |  |  |  |  |  |  |
| 250-500 bp | mean |  | 53.47 |  |  |  |
|  | median |  | 61.00 |  |  |  |
|  | range |  | 16.11-83.84 |  |  |  |
|  |  |  |  |  |  |  |
| 500-10 380 bp | mean |  | 88.18 |  |  |  |
|  | median |  | 66.10 |  |  |  |
|  | range |  | 20.76-166.56 |  |  |  |
|  | Donation at the time of ongoing carcinogenesis at different tumor location and lesion of the disease [pg/μl ] |  |  |  |  |  |
|  | GROUP I |  | N=9.00 |  |  |  |
| 35-250 bp | median PTu |  | 220.25 |  |  |  |
|  | median RTu |  | 236.44 |  |  |  |
|  |  |  |  |  |  |  |
| 35-1 000 bp | median PTu |  | 301.50 |  |  |  |
|  | median RTu |  | 319.86 |  |  |  |
|  |  |  |  |  |  |  |
| 35-10 380 bp | median PTu |  | 351.75 |  |  |  |
|  | median RTu |  | 382.24 |  |  |  |
|  |  |  |  |  |  |  |
| 250-500 bp | median PTu |  | 48.16 |  |  |  |
|  | median RTu |  | 54.57 |  |  |  |
|  |  |  |  |  |  |  |
| 500-10 380 bp | median PTu |  | 54.10 |  |  |  |
|  | median RTu |  | 91.24 |  |  |  |
|  |  |  |  |  |  |  |
|  | GROUP II |  | N=43.00 |  |  |  |
| 35-250 bp | median PTu |  | 371.43 |  |  |  |
|  | median RTu |  | 279.31 |  |  |  |
|  |  |  |  |  |  |  |
| 35-1 000 bp | median PTu |  | 480.35 |  |  |  |
|  | median RTu |  | 379.89 |  |  |  |
|  |  |  |  |  |  |  |
| 35-10 380 bp | median PTu |  | 581.07 |  |  |  |
|  | median RTu |  | 422.76 |  |  |  |
|  |  |  |  |  |  |  |
| 250-500 bp | median PTu |  | 71.50 |  |  |  |
|  | median RTu |  | 66.28 |  |  |  |
|  |  |  |  |  |  |  |
| 500-10 380 bp | median PTu |  | 88.48 |  |  |  |
|  | median RTu |  | 54.22 |  |  |  |
|  |  |  |  |  |  |  |
|  | GROUP IV |  | N=6.00 |  |  |  |
| 35-250 bp | median PTu |  | 248.35 |  |  |  |
|  | median RTu |  | 283.07 |  |  |  |
|  |  |  |  |  |  |  |
| 35-1 000 bp | median PTu |  | 329.42 |  |  |  |
|  | median RTu |  | 369.88 |  |  |  |
|  |  |  |  |  |  |  |
| 35-10 380 bp | median PTu |  | 384.22 |  |  |  |
|  | median RTu |  | 404.24 |  |  |  |
|  |  |  |  |  |  |  |
| 250-500 bp | median PTu |  | 61.18 |  |  |  |
|  | median RTu |  | 59.60 |  |  |  |
|  |  |  |  |  |  |  |
| 500-10 380 bp | median PTu |  | 79.76 |  |  |  |
|  | median RTu |  | 61.57 |  |  |  |
|  |  |  |  |  |  |  |
|  | GROUP V |  | N=101.00 |  |  |  |
| 35-250 bp | median PTu |  | 260.21 |  |  |  |
|  | median RTu |  | 236.27 |  |  |  |
|  |  |  |  |  |  |  |
| 35-1 000 bp | median PTu |  | 342.96 |  |  |  |
|  | median RTu |  | 354.33 |  |  |  |
|  |  |  |  |  |  |  |
| 35-10 380 bp | median PTu |  | 394.20 |  |  |  |
|  | median RTu |  | 426.62 |  |  |  |
|  |  |  |  |  |  |  |
| 250-500 bp | median PTu |  | 47.14 |  |  |  |
|  | median RTu |  | 60.55 |  |  |  |
|  |  |  |  |  |  |  |
| 500-10 380 bp | median PTu |  | 80.07 |  |  |  |
|  | median RTu |  | 102.52 |  |  |  |
|  |  |  |  |  |  |  |
|  | GROUP VI |  | N=6.00 |  |  |  |
| 35-250 bp | median PTu |  | 230.40 |  |  |  |
|  | median RTu |  | 247.07 |  |  |  |
|  |  |  |  |  |  |  |
| 35-1 000 bp | median PTu |  | 329.33 |  |  |  |
|  | median RTu |  | 340.87 |  |  |  |
|  |  |  |  |  |  |  |
| 35-10 380 bp | median PTu |  | 381.25 |  |  |  |
|  | median RTu |  | 384.25 |  |  |  |
|  |  |  |  |  |  |  |
| 250-500 bp | median PTu |  | 47.93 |  |  |  |
|  | median RTu |  | 62.50 |  |  |  |
|  |  |  |  |  |  |  |
| 500-10 380 bp | median PTu |  | 64.23 |  |  |  |
|  | median RTu |  | 106.66 |  |  |  |
|  |  |  |  |  |  |  |
|  | Donation at the time of ongoing carcinogenesis at different tumor location including T parameter [pg/μl ] |  |  |  |  |  |
|  | GROUP I |  | N=9.00 |  |  |  |
| 35-250 bp | median T1/T2 |  | 232.47 |  |  |  |
|  | median T3/T4 |  | 165.57 |  |  |  |
|  |  |  |  |  |  |  |
| 35-1 000 bp | median T1/T2 |  | 331.15 |  |  |  |
|  | median T3/T4 |  | 237.67 |  |  |  |
|  |  |  |  |  |  |  |
| 35-10 380 bp | median T1/T2 |  | 378.53 |  |  |  |
|  | median T3/T4 |  | 290.85 |  |  |  |
|  |  |  |  |  |  |  |
| 250-500 bp | median T1/T2 |  | 75.48 |  |  |  |
|  | median T3/T4 |  | 45.04 |  |  |  |
|  |  |  |  |  |  |  |
| 500-10 380 bp | median T1/T2 |  | 76.24 |  |  |  |
|  | median T3/T4 |  | 62.28 |  |  |  |
|  |  |  |  |  |  |  |
|  | GROUP II |  | N=43.00 |  |  |  |
| 35-250 bp | median T1/T2 |  | 373.19 |  |  |  |
|  | median T3/T4 |  | 358.88 |  |  |  |
|  |  |  |  |  |  |  |
| 35-1 000 bp | median T1/T2 |  | 492.97 |  |  |  |
|  | median T3/T4 |  | 511.91 |  |  |  |
|  |  |  |  |  |  |  |
| 35-10 380 bp | median T1/T2 |  | 531.39 |  |  |  |
|  | median T3/T4 |  | 660.49 |  |  |  |
|  |  |  |  |  |  |  |
| 250-500 bp | median T1/T2 |  | 66.12 |  |  |  |
|  | median T3/T4 |  | 86.41 |  |  |  |
|  |  |  |  |  |  |  |
| 500-10 380 bp | median T1/T2 |  | 76.05 |  |  |  |
|  | median T3/T4 |  | 105.47 |  |  |  |
|  |  |  |  |  |  |  |
|  | GROUP IV |  | N=6.00 |  |  |  |
| 35-250 bp | median T1/T2 |  | 196.90 |  |  |  |
|  | median T3/T4 |  | 270.88 |  |  |  |
|  |  |  |  |  |  |  |
| 35-1 000 bp | median T1/T2 |  | 283.04 |  |  |  |
|  | median T3/T4 |  | 365.57 |  |  |  |
|  |  |  |  |  |  |  |
| 35-10 380 bp | median T1/T2 |  | 305.56 |  |  |  |
|  | median T3/T4 |  | 429.92 |  |  |  |
|  |  |  |  |  |  |  |
| 250-500 bp | median T1/T2 |  | 58.93 |  |  |  |
|  | median T3/T4 |  | 61.85 |  |  |  |
|  |  |  |  |  |  |  |
| 500-10 380 bp | median T1/T2 |  | 49.72 |  |  |  |
|  | median T3/T4 |  | 83.30 |  |  |  |
|  |  |  |  |  |  |  |
|  | GROUP V |  | N=101.00 |  |  |  |
| 35-250 bp | median T1/T2 |  | 243.57 |  |  |  |
|  | median T3/T4 |  | 296.07 |  |  |  |
|  |  |  |  |  |  |  |
| 35-1 000 bp | median T1/T2 |  | 316.80 |  |  |  |
|  | median T3/T4 |  | 352.60 |  |  |  |
|  |  |  |  |  |  |  |
| 35-10 380 bp | median T1/T2 |  | 358.62 |  |  |  |
|  | median T3/T4 |  | 411.68 |  |  |  |
|  |  |  |  |  |  |  |
| 250-500 bp | median T1/T2 |  | 42.49 |  |  |  |
|  | median T3/T4 |  | 60.31 |  |  |  |
|  |  |  |  |  |  |  |
| 500-10 380 bp | median T1/T2 |  | 79.27 |  |  |  |
|  | median T3/T4 |  | 87.00 |  |  |  |
|  |  |  |  |  |  |  |
|  | GROUP VI |  | N=6.00 |  |  |  |
| 35-250 bp | median T1/T2 |  | 230.40 |  |  |  |
|  | median T3/T4 |  | 247.07 |  |  |  |
|  |  |  |  |  |  |  |
| 35-1 000 bp | median T1/T2 |  | 329.33 |  |  |  |
|  | median T3/T4 |  | 340.87 |  |  |  |
|  |  |  |  |  |  |  |
| 35-10 380 bp | median T1/T2 |  | 381.25 |  |  |  |
|  | median T3/T4 |  | 384.25 |  |  |  |
|  |  |  |  |  |  |  |
| 250-500 bp | median T1/T2 |  | 47.93 |  |  |  |
|  | median T3/T4 |  | 62.50 |  |  |  |
|  |  |  |  |  |  |  |
| 500-10 380 bp | median T1/T2 |  | 64.23 |  |  |  |
|  | median T3/T4 |  | 106.66 |  |  |  |
|  |  |  |  |  |  |  |
|  |  |  |  |  |  |  |

| Table S3 HNSCC patients characteristics concerning cfDNA length | | | |
| --- | --- | --- | --- |
|  |  |  |  |
|  | Donation at the time of ongoing carcinogenesis [bp] |  | N=166 |
| 35-250 bp | mean |  | 153.32 |
|  | median |  | 156.00 |
|  | range |  | 93-181 |
|  |  |  |  |
| 35-1 000 bp | mean |  | 275.06 |
|  | median |  | 270.25 |
|  | range |  | 144-479 |
|  |  |  |  |
| 500-10 380 bp | mean |  | 4 765.54 |
|  | median |  | 4 589.00 |
|  | range |  | 2 571 - 9 387 |
|  |  |  |  |
| 35-10 380 bp | mean |  | 2 210.30 |
|  | median |  | 2 154.50 |
|  | range |  | 436-4 282 |
|  |  |  |  |
|  | Healthy controls [bp] |  | N=56 |
| 35-250 bp | mean |  | 147.64 |
|  | median |  | 149.00 |
|  | range |  | 100-174 |
|  |  |  |  |
| 35-1 000 bp | mean |  | 305.75 |
|  | median |  | 304.00 |
|  | range |  | 212-430 |
|  |  |  |  |
| 500-10 380 bp | mean |  | 4 817.92 |
|  | median |  | 4 691.33 |
|  | range |  | 2 887.33-7 169.5 |
|  |  |  |  |
| 35-10 380 bp | mean |  | 2 542.77 |
|  | median |  | 2 355.00 |
|  | range |  | 1 074-4 235 |
|  |  |  |  |
|  | Donation at the time of ongoing carcinogenesis PTu [bp] |  | N=137 |
| 35-250 bp | mean |  | 152.75 |
|  | median |  | 156.00 |
|  | range |  | 93-181 |
|  |  |  |  |
| 35-1 000 bp | mean |  | 274.12 |
|  | median |  | 268.00 |
|  | range |  | 144-479 |
|  |  |  |  |
| 500-10 380 bp | mean |  | 4 850.60 |
|  | median |  | 4 679.00 |
|  | range |  | 2 571-9 387 |
|  |  |  |  |
| 35-10 380 bp | mean |  | 2 260.98 |
|  | median |  | 2 223.50 |
|  | range |  | 436-4 282 |
|  |  |  |  |
|  | Donation at the time of ongoing carcinogenesis RTu [bp] |  | N=29 |
| 35-250 bp | mean |  | 156.02 |
|  | median |  | 159.00 |
|  | range |  | 127-173 |
|  |  |  |  |
| 35-1 000 bp | mean |  | 279.48 |
|  | median |  | 280.00 |
|  | range |  | 196-341 |
|  |  |  |  |
| 500-10 380 bp | mean |  | 4 366.26 |
|  | median |  | 4 245.00 |
|  | range |  | 3 125.50-6 671 |
|  |  |  |  |
| 35-10 380 bp | mean |  | 1 954.25 |
|  | median |  | 1 861.50 |
|  | range |  | 749-3 762.5 |
|  |  |  |  |
|  | First donation from patients without recurrence [bp] |  | N=114 |
| 35-250 bp | mean |  | 153.25 |
|  | median |  | 156.00 |
|  | range |  | 93-181 |
|  |  |  |  |
| 35-1 000 bp | mean |  | 273.37 |
|  | median |  | 268.25 |
|  | range |  | 144-462 |
|  |  |  |  |
| 500-10 380 bp | mean |  | 4 832.51 |
|  | median |  | 4 598.50 |
|  | range |  | 2 821-9 387 |
|  |  |  |  |
| 35-10 380 bp | mean |  | 2 254.16 |
|  | median |  | 2 252.25 |
|  | range |  | 436-4 282 |
|  |  |  |  |
|  | First donation at the time of primary tumor diagnosis from patients with recurrence in the follow-up [bp] |  | N=19 |
| 35-250 bp | mean |  | 148.11 |
|  | median |  | 151.00 |
|  | range |  | 125-167 |
|  |  |  |  |
| 35-1 000 bp | mean |  | 279.50 |
|  | median |  | 249.00 |
|  | range |  | 208-479 |
|  |  |  |  |
| 500-10 380 bp | mean |  | 5 034.34 |
|  | median |  | 5 302.00 |
|  | range |  | 2 571-6 442 |
|  |  |  |  |
| 35-10 380 bp | mean |  | 2 383.84 |
|  | median |  | 2 328.00 |
|  | range |  | 953-3 830 |


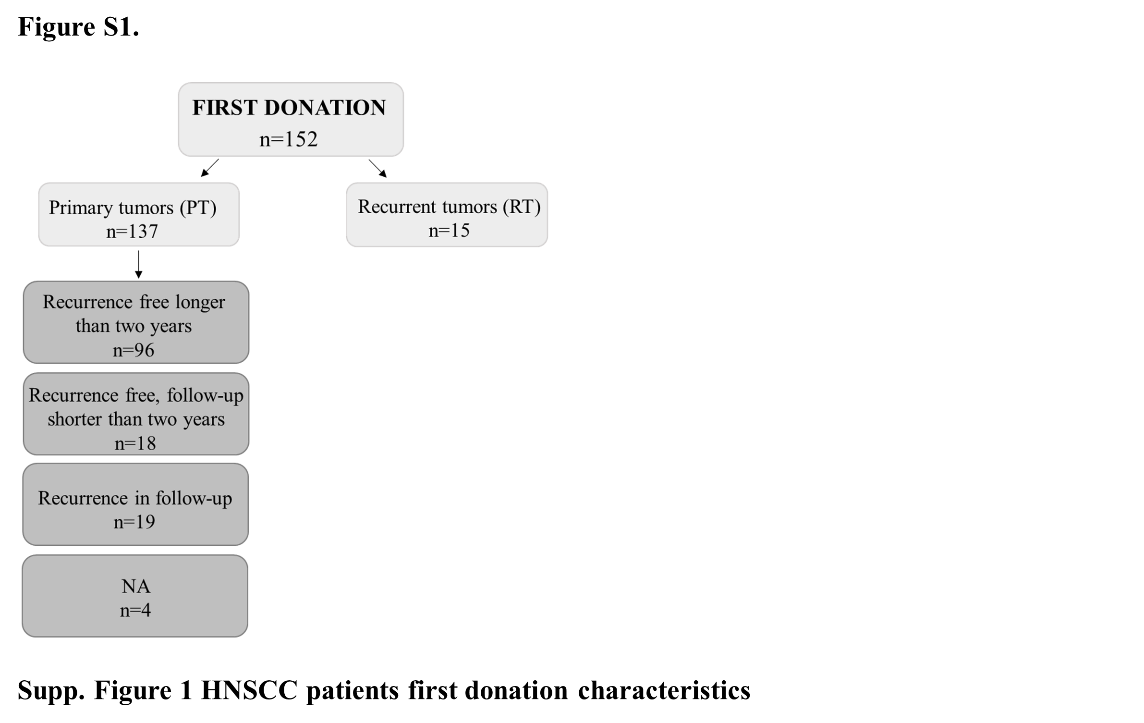


**Figure S1. HNSCC patients first donation characteristics concerning the course of disease. The course of disease of 4 patients was unclear therefore they were marked as “NA” and excluded from further study.**

**
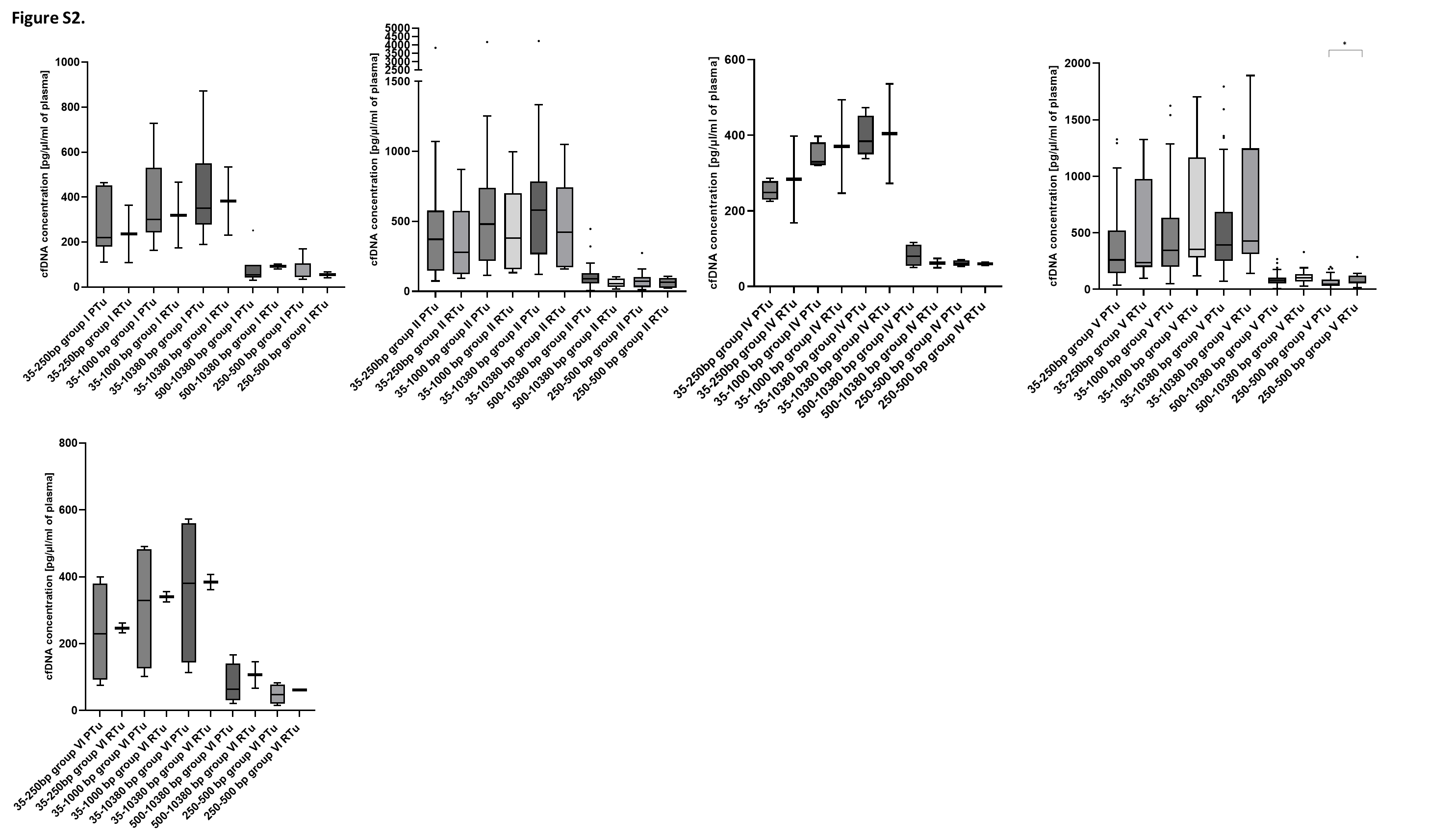
**

**Figure S2. Concentration of cfDNA in different ranges of length, depending on tumor location and the course of disease.** Group 1 (I) PTu (n=7), RTu (n=2);
group 2 (II) PTu (n=37), RTu (n=6); group 4 (IV) PTu (n=4), RTu (n=2); group 5 (V) PTu (n=84), RTu (n=17); group 6 (VI) PTu (n=4), RTu (n=2). Kruskal-Wallis Rank Sum Test followed by post hoc Dunn’s test was performed.


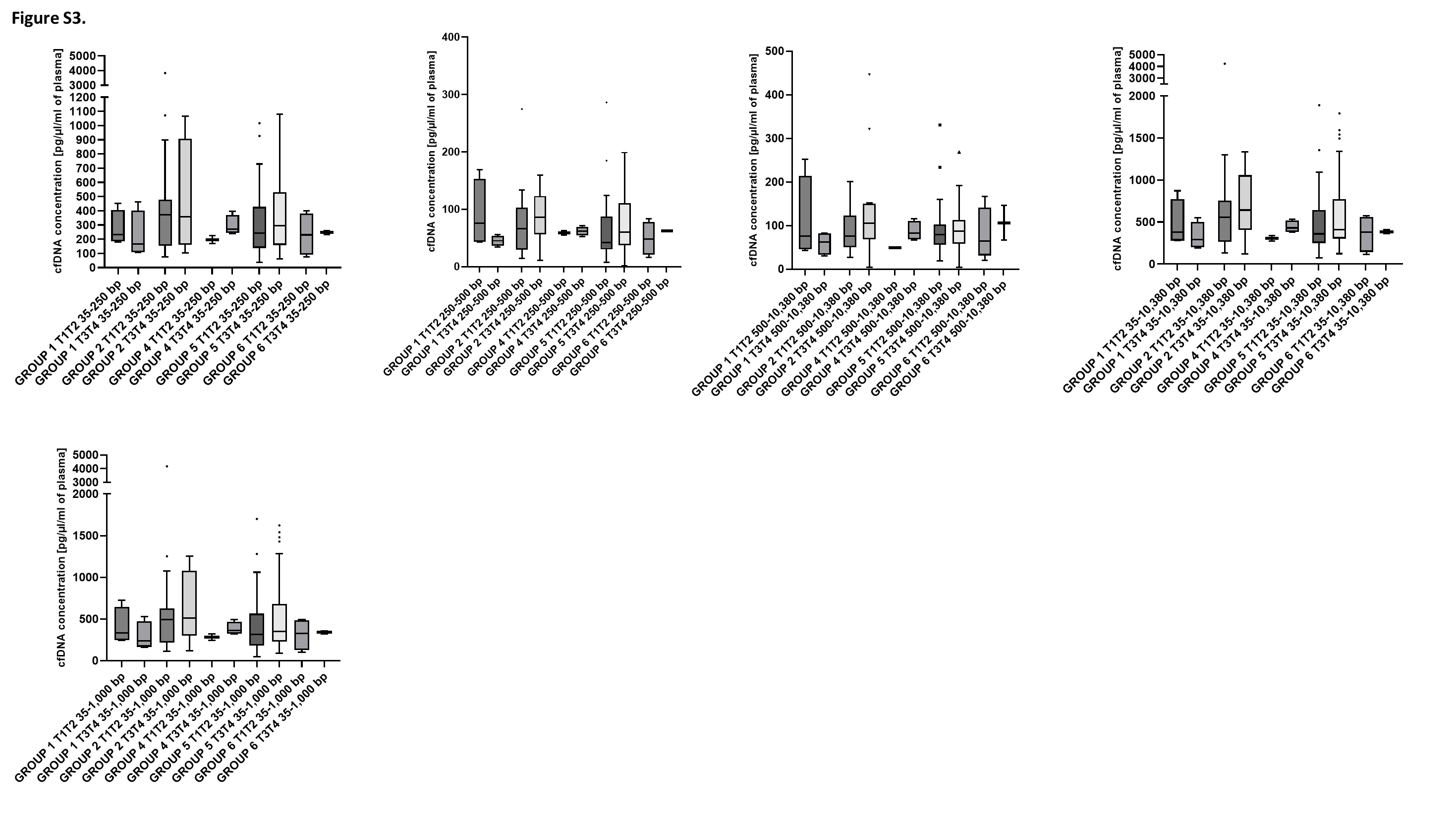


**Figure S3. Concentration of cfDNA in different ranges of length, depending on tumor location and T parameter (T1/T2 and T3/T4) according to the TNM classification.** Group 1 T1/T2 (n=4), T3/T4 (n=4), NA (n=1); group 2 T1/T2 (n=28), T3/T4 (n=12), NA (n=3); group 4 T1/T2 (n=2), T3/T4 (n=4); group 5 T1/T2 (n=45), T3/T4 (n=56);
group 6 T1/T2 (n=4), T3/T4 (n=2). Kruskal-Wallis Rank Sum Test followed by post hoc Dunn’s test was performed.
